# CDK4/6i-treated HR+/HER2- breast cancer tumors show higher ESR1 mutation prevalence and more altered genomic landscape

**DOI:** 10.1038/s41523-024-00617-7

**Published:** 2024-02-22

**Authors:** Nayan Chaudhary, Alejandro M. Chibly, Ann Collier, Jorge Martinalbo, Pablo Perez-Moreno, Heather M. Moore, Patricia Luhn, Ciara Metcalfe, Marc Hafner

**Affiliations:** 1 Real World Data Science, Genentech Inc., South San Francisco, CA USA; 2 Department of Oncology Bioinformatics, Genentech Inc., South San Francisco, CA USA; 3 Department of Translational Medicine Oncology, Genentech Inc., South San Francisco, CA USA; 4 Department of Product Development Oncology, Hoffmann La Roche, Basel, Switzerland; 5 Department of Clinical Development Oncology, Genentech Inc., South San Francisco, CA USA; 6 Department of Discovery Oncology, Genentech Inc., South San Francisco, CA USA

**Keywords:** Cancer genomics, Breast cancer, Oncogenes

## Abstract

As CDK4/6 inhibitor (CDK4/6i) approval changed treatment strategies for patients with hormone receptor-positive HER2-negative (HR+/HER2-) breast cancer (BC), understanding how exposure to CDK4/6i affects the tumor genomic landscape is critical for precision oncology. Using real-world data (RWD) with tumor genomic profiling from 5910 patients with metastatic HR+/HER2- BC, we investigated the evolution of alteration prevalence in commonly mutated genes across patient journeys. We found that *ESR1* is more often altered in tumors exposed to at least 1 year of adjuvant endocrine therapy, contrasting with *TP53* alterations. We observed a similar trend after first-line treatments in the advanced setting, but strikingly exposure to aromatase inhibitors (AI) combined with CDK4/6i led to significantly higher *ESR1* alteration prevalence compared to AI alone, independent of treatment duration. Further, CDK4/6i exposure was associated with higher occurrence of concomitant alterations in multiple oncogenic pathways. Differences based on CDK4/6i exposure were confirmed in samples collected after 2L and validated in samples from the acelERA BC clinical trial. In conclusion, our work uncovers opportunities for further treatment personalization and stresses the need for effective combination treatments to address the altered tumor genomic landscape following AI+CDK4/6i exposure. Further, we demonstrated the potential of RWD for refining patient treatment strategy and guiding clinical trial design.

## Introduction

The first publicly available large genomic datasets from tumor samples have yielded numerous discoveries and continue to be reference datasets for cancer research^1–3^. Since then, other studies have characterized the genomic landscape of metastatic breast cancers (mBC)^4–13^, and datasets comprising patient clinical history and tumor genomics data are becoming more widely available. Multiple efforts are ongoing to collect, curate, and publish such clinico-genomic datasets, including the AACR project GENIE^14^, MSK-IMPACT study^15^, plasmaMATCH^16^, or POG570^17^. Studies with clinico-genomics data led to the identification of resistance mechanisms, in particular *ESR1* mutations, which are associated with disease progression on aromatase inhibitors (AI) in hormone receptor-positive HER2-negative (HR+/HER2-) breast cancers (BC)^15–22^. The wide use of CDK4/6 inhibitors (CDK4/6i) for treating HR+/HER2- BC^23,24^ raised the question of identifying alterations associated with de novo and acquired resistance to CDK4/6i^25–28^ and more generally of how the genomic landscape of CDK4/6i-naive tumors differ from those exposed to CDK4/6i^29–31^. Understanding these differences is critical not only for adapting treatment strategies following CDK4/6i exposure^32^ but also for designing and interpreting clinical trials for novel targeted therapies whose clinical benefit compared to current standards of care may be higher for tumors harboring specific mutations such as *ESR1* for oral selective estrogen receptor antagonists and degraders (SERD)^33,34^ or *PIK3CA* and *AKT1* for PI3K/AKT pathway inhibitors^35^.

To systematically characterize how the genomic profiles of HR+/HER2- tumors evolve across lines of therapies, we selected a cohort of 5910 patients with mBC leveraging a real-world dataset (RWD) from a nationwide de-identified clinico-genomic database which has linked electronic health records (EHRs) and comprehensive genomic profiling (CGP). Similar data have been used to recover known associations of patients and tumor characteristics with clinical outcomes in lung cancer^36^ and other diseases^37^, as well as comparing clinical trial patients to those in community practice^38^. While RWD may be more representative of the overall patient population^39^, their use for exploratory analyses requires specific considerations. Here, we aimed at assessing differences in the prevalence of genomic alterations between unpaired samples at different stages of the treatment continuum. In addition to previous studies^10,31^, we performed stratified analyses based on potential confounders of tumor genomic profile such as the site of the collected samples or patient clinical history to exclude biases and spurious results due to unbalanced groups. After studying the impact of the duration of adjuvant therapy on prevalence of alterations in *TP53*, which is prognostic^40^, and *ESR1*, which is associated with AI-resistance, we evaluated changes in the tumor genomic landscape following first-line (1L) treatment and in later lines (2L+). We found significant differences in alteration prevalence in tumors exposed to AI+CDK4/6i relative to those exposed only to AI. The results based on RWD were validated in data from a recent clinical trial, acelERA BC^41^. Overall, the differences we found in the tumor genomic landscape suggest that treatment strategies and the development of new therapeutics for advanced HR+/HER2- BC may need to be adapted for patients previously treated with CDK4/6i.

## Results

### Database and cohort selection

**Fig. 1:**
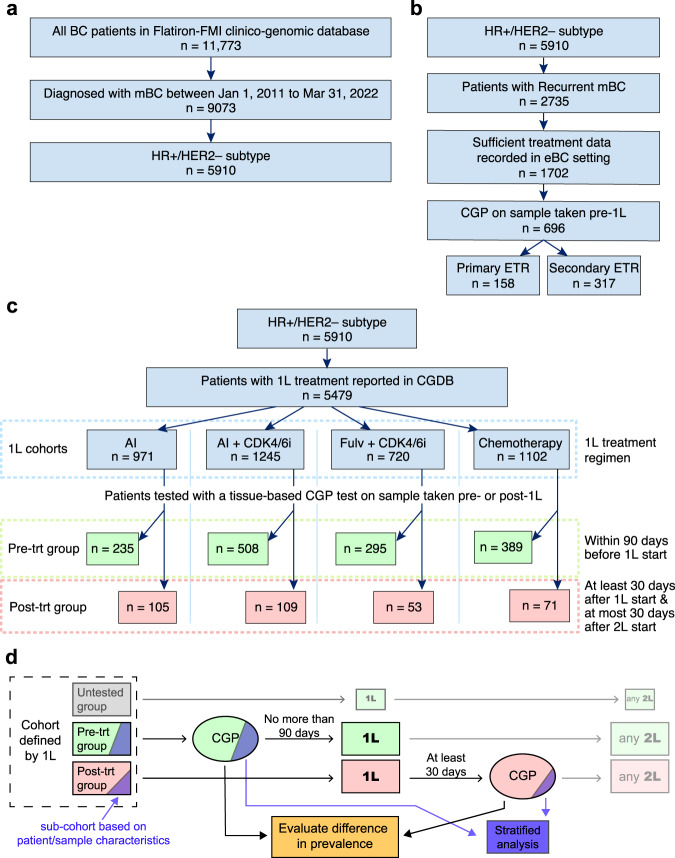
Cohort selection and study design. **a** Selection of patients with HR+/HER2- BC in the CGDB. **b** Attrition of the eBC cohorts in our study. **c** Attrition of the 1L cohorts in our study and split into pre- and post-1L groups based on the line of treatment and timing of the sample collection for CGP testing. **d** Patients are selected based on their 1L regimen and are divided into subgroups based on the timing of their CGP. The effect of treatment on gene alterations is estimated by comparing alteration prevalence in tumors profiled prior to the 1L versus tumors profiled after 1L. Stratified analyses are based on a stratum of patients defined by clinical variables (e.g. de novo vs. recurrent disease) or sample characteristics. eBC/mBC stands for early/metastatic breast cancer, HR+/HER2- for hormone-receptor positive HER2-negative, CGP for comprehensive genomic profiling, 1L for first-line treatment, ETR for endocrine treatment resistance, CGDB for clinico-genomic database, AI for aromatase inhibitor, Fulv for fulvestrant.

This study relied on the nationwide (US-based) de-identified Flatiron Health-Foundation Medicine Inc (FH-FMI) clinico-genomic database (CGDB). The CGDB has been described and validated in previous publications^10,36,37,42^. Details for patient inclusion are described in the Methods section. In this work, we focused on a cohort of 5910 patients who were diagnosed with HR+/HER2- mBC (see Fig. 1a and Methods) and had confirmed metastatic diagnosis between Jan 1, 2011 and March 31, 2022 (database cutoff date).

Among those 5910 patients, we defined multiple cohorts based on the line of treatment. First, to study the effects of adjuvant therapy on the tumor genomic landscape, we built a cohort comprising 1702 patients with recurrent mBC who had sufficient treatment information recorded in the FH database during their early breast cancer (eBC) disease (Fig. 1b) to evaluate their endocrine resistance status.

We classified patients as either primary (<2 years on adjuvant ET) or secondary (>2 years on adjuvant ET) endocrine resistant, guided by the ESMO definition for endocrine treatment resistance (ETR), with both groups relapsing within 1 year from last ET.

Second, we defined first line cohorts based on the 1L advanced treatment regimen: aromatase inhibitors (AI) alone or in combination with CDK4/6 inhibitors (AI+CDK4/6i), fulvestrant with CDK4/6i (Fulv+CDK4/6i), or chemotherapies (any regimen without endocrine or targeted therapies). Other 1L treatment regimens such as fulvestrant alone, SERM-based regimens (specific estrogen receptor modulators), CDK4/6i alone, PI3Ki-based regimens, or combination of multiple therapies had too few patients with CGP (≤ 30 patients post-1L) to enable meaningful analyses. Then, we defined our pre- and post-treatment groups for the 1L cohort based on the *sample collection date* for the CGP. For the pre-1L group, we included patients with a sample collected at most 90 days before the start of 1L. For the post-1L group, we included patients whose samples were collected at least 30 days after the start of 1L of therapy and at most 30 days after the start of 2 L of therapy (Fig. 1c, d). Patient and sample characteristics for each cohort are reported in Table 1.

**Table 1:** Baseline demographic, clinical and tumor characteristics for the first-line patient cohorts.

	AI		AI+CDK4/6i		Fulv+CDK4/6i		Chemotherapy	
	Pre trt. (*n* = 235)	Post trt. (*n* = 105)	Pre trt. (*n* = 508)	Post trt. (*n* = 109)	Pre trt. (*n* = 295)	Post trt. (*n* = 53)	Pre trt. (*n* = 389)	Post trt. (*n* = 71)
**Age at sample collection**								
Mean (SD)	64.0 (11.5)	63.9 (11.9)	61.1 (11.3)	60.0 (10.9)	62.6 (10.6)	61.8 (10.7)	55.9 (12.4)	59.1 (11.9)
Median [Min, Max]	65.0 [30.0, 85.0]	65.0 [36.0, 84.0]	63.0 [27.0, 84.0]	61.0 [29.0, 83.0]	63.0 [32.0, 83.0]	62.0 [31.0, 80.0]	57.0 [28.0, 82.0]	60.0 [29.0, 80.0]
**Race**								
African American	16 (6.8%)	7 (6.7%)	43 (8.5%)	9 (8.3%)	19 (6.4%)	≤5	36 (9.3%)	8 (11.3%)
White	166 (70.6%)	68 (64.8%)	339 (66.7%)	78 (71.6%)	220 (74.6%)	39 (73.6%)	256 (65.8%)	48 (67.6%)
Other*	53 (22.6%)	30 (28.6%)	126 (24.8%)	22 (20.2%)	56 (19.0%)	10 (18.9%)	97 (24.9%)	15 (21.1%)
**Stage at met diagnosis**								
De novo	99 (42.1%)	21 (20.0%)	257 (50.6%)	44 (40.4%)	33 (11.2%)	7 (13.2%)	117 (30.1%)	15 (21.1%)
Recurrent	136 (57.9%)	84 (80.0%)	251 (49.4%)	65 (59.6%)	262 (88.8%)	46 (86.8%)	272 (69.9%)	56 (78.9%)
**Sample collection site**								
Primary	76 (32.3%)	16 (15.2%)	176 (34.6%)	23 (21.1%)	36 (12.2%)	6 (11.3%)	129 (33.2%)	13 (18.3%)
Metastatic	159 (67.7%)	89 (84.8%)	332 (65.4%)	86 (78.9%)	259 (87.8%)	47 (88.7%)	260 (66.8%)	58 (81.7%)
**Tissue of origin**								
Breast	76 (32.3%)	16 (15.2%)	176 (34.6%)	23 (21.1%)	36 (12.2%)	6 (11.3%)	129 (33.2%)	13 (18.3%)
Bone	29 (12.3%)	11 (10.5%)	74 (14.6%)	13 (11.9%)	50 (16.9%)	≤5	22 (5.7%)	6 (8.5%)
Liver	15 (6.4%)	18 (17.1%)	49 (9.6%)	33 (30.3%)	72 (24.4%)	22 (41.5%)	89 (22.9%)	18 (25.4%)
Lung	13 (5.5%)	8 (7.6%)	39 (7.7%)	≤5	18 (6.1%)	≤5	19 (4.9%)	≤5
Lymph node	23 (9.8%)	16 (15.2%)	51 (10.0%)	9 (8.3%)	32 (10.8%)	≤5	40 (10.3%)	10 (14.1%)
Soft tissue	17 (7.2%)	8 (7.6%)	20 (3.9%)	≤5	16 (5.4%)	≤5	14 (3.6%)	6 (8.5%)
Other	62 (26.4%)	28 (26.7%)	99 (19.5%)	24 (22.0%)	71 (24.1%)	13 (24.5%)	76 (19.5%)	13 (18.3%)
**ECOG**^**†**^ **performance status at sample collection**								
0	46 (19.6%)	39 (37.1%)	144 (28.3%)	37 (33.9%)	121 (41.0%)	23 (43.4%)	117 (30.1%)	27 (38.0%)
1	38 (16.2%)	30 (28.6%)	75 (14.8%)	39 (35.8%)	70 (23.7%)	18 (34.0%)	59 (15.2%)	21 (29.6%)
≥2	9 (3.8%)	10 (9.5%)	12 (2.4%)	6 (5.5%)	12 (4.1%)	6 (11.3%)	13 (3.3%)	≤5
Missing	142 (60.4%)	26 (24.8%)	277 (54.5%)	27 (24.8%)	92 (31.2%)	6 (11.3%)	200 (51.4%)	21 (29.6%)
**Visceral disease at sample collection**								
Yes	60 (25.5%)	39 (37.1%)	146 (28.7%)	59 (54.1%)	94 (31.9%)	35 (66.0%)	166 (42.7%)	38 (53.5%)
No	175 (74.5%)	66 (62.9%)	362 (71.3%)	50 (45.9%)	201 (68.1%)	18 (34.0%)	223 (57.3%)	33 (46.5%)

*Includes Unknown and missing values.

^†^Eastern Cooperative Oncology Group.

Last, we defined later line patient cohorts by selecting patients who had prior exposure to AI in the advanced setting (representing 78% of samples collected after 2L) and splitting them based on prior exposure to either CDK4/6i or chemotherapies independently of exposure to any endocrine therapies. We removed patients who were exposed to other targeted therapies such as PI3K or mTOR inhibitors. We further split these cohorts into two groups: samples collected at least 30 days after the start of the second and no more than 30 days after the start of the fourth line (2–3L) and those collected at least 30 days after the start of the fourth line (4L+). Demographic and sample properties for each cohort are reported in Supplementary Table 1. For the patients who provided multiple samples, we considered the samples independently based on their time of collection and prior treatments.

### Adjuvant therapy duration is associated with differences in tumor genomic profiles

**Fig. 2:**
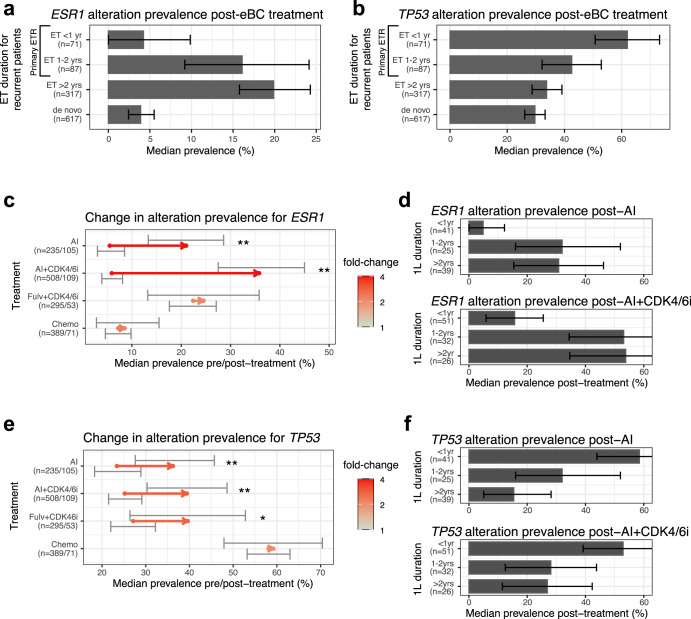
Prevalence of *ESR1* and *TP53* alterations in advanced HR+ breast cancer tumors is associated with treatment duration and CDK4/6i exposure. **a**, **b** Prevalence of *ESR1* (**a**) and *TP53* (**b**) alterations in samples collected from patients prior to 1L split by duration of eBC ET (endocrine treatment) prior to relapse. Error bars represent the 95% confidence interval based on bootstrapping. **c** Prevalence of *ESR1* alterations prior or after 1L treatment in the advanced setting in different cohorts. Arrow represents the difference in prevalence: its origin is the median prevalence pre-treatment and its end is the median prevalence post-treatment. Error bars represent the 95% confidence interval of prevalence; Color represents fold-change magnitude; * stands for *p* < 0.05, ** for *p* < 0.01 based on bootstrapping; Treatment cohorts are labeled on the *y*-axis. **d** Prevalence of *ESR1* alterations after 1L treatment stratified by the 1L treatment duration prior to sample collection. Error bars represent the 95% confidence interval of prevalence. **e** Prevalence of *TP53* alterations prior or after 1L treatment in the advanced setting in different cohorts. Same legend as **c**. **f** Prevalence of *TP53* alterations after 1L treatment stratified by the 1L treatment duration prior to sample collection. Same legend as **d**.

We started by characterizing the genomic landscape of tumors from endocrine resistant patients using CGP data from samples collected after the adjuvant treatment and prior to the initiation of 1L treatments. We found that *ESR1* alteration prevalence was significantly higher in tumors from patients with secondary ETR (19.9% [CI: 15.6, 24.7]) compared to those from patients with primary ETR (10.7% [CI: 6.3–15.8], *p* < 10^−4^) (Supplementary Fig. 1a). In contrast, *TP53* alterations were significantly more prevalent in primary ETR samples (51.3% [CI: 43.7–58.9]) compared to secondary ETR samples (33.8% [CI: 28.4–39.1], *p* < 10^−4^, Supplementary Fig. 1b). Other genes had smaller differences between the two groups of patients with ETR (Supplementary Fig. 1c). By assessing changes in prevalence based on the duration of adjuvant endocrine treatment (ET) before relapse, we found that the genomic profile of tumors transitioned around a 1-year cutoff. Alteration prevalence for tumors exposed to 1–2 years of ET was 16.1% (CI: 9.2–24.1) for *ESR1* (Fig. 2a) and 42.5% (CI: 32.2–52.9) for *TP53* (Fig. 2b)—values that are closer to the prevalence found in secondary ETR samples than samples exposed to less than 1 year of adjuvant ET (4.2% [CI: 0.0–9.9] and 62.0% [CI: 50.7–73.2], respectively). Other genes showed further separation when using 1 year as a cutoff for adjuvant ET duration before relapse (Supplementary Fig. 1d). Compared to the prevalence of alterations found in samples from patients with de novo metastatic disease (3.9% [CI: 2.4–5.5] for *ESR1* and 29.7% [CI: 26.1–33.2] for *TP53*), tumors from patients relapsing within 1 year on adjuvant ET were characterized by substantially higher prevalence of *TP53* alterations, whereas tumors from other recurrent patients (>1 years on adjuvant ET) had higher *ESR1* alteration prevalence (19.1% [CI: 15.3–22.8]).

### ESR1 alteration prevalence post-AI+CDK4/6i is higher than post-AI

Next, we studied the interaction between 1L treatments and tumor genomic profiles by comparing the alteration prevalence in samples taken prior to 1L to those taken after 1L exposure (Fig. 1d, see Methods). As expected, we observed an increase of *ESR1* alterations following AI-based treatments. Compared to other studies^8–10,16,17,19^, we split patients based on CDK4/6i exposure, which identified a significant difference (Fig. 2c): the prevalence of *ESR1* alterations was increased substantially more in samples from patients treated with AI+CDK4/6i (35.8% [CI: 27.5-45.0]; *p* < 10^−4^) compared to those treated with AI alone (21.0% [CI: 13.3–28.6], *p* < 10^−4^), resulting in 1.71-fold difference (CI: 1.11–2.81, *p* = 0.0066) post-treatment. The prevalence in pre-treatment groups was similar at 5.5% (CI: 3.0–8.5) and 5.9% (CI: 3.9–8.1) for AI and AI+CDK4/6i cohorts, respectively. In contrast, the cohort of patients treated with fulvestrant+CDK4/6i showed a minimal increase from 22.4% (CI: 17.6–27.1) pre-treatment to 24.5% (CI: 13.2–35.8) post-treatment (*p* = 0.38). The higher prevalence of *ESR1* alteration pre-treatment in this cohort may be explained by a higher proportion of patients with recurrent disease compared to other 1L cohorts (Table 1). The non-significant increase in post-treatment *ESR1* alteration prevalence was consistent with the PALOMA-3 trial^43^. The chemotherapy cohort also showed no difference in the prevalence of *ESR1* alterations between the pre- and post-treatment groups (*p* = 0.38). For the large majority of patients, a single subclonal variant was detected in the tumor and the distribution of cumulative allele frequencies was not substantially different between groups and treatment cohorts (Supplementary Fig. 2a) with the more than 75% of the mutations being at codons L536, Y537, or D538.

To validate the difference in *ESR1* alteration prevalence post-treatment based on CDK4/6i exposure, we performed stratified analyses (Fig. 1d, see Methods). First, we found that the difference was not associated with the duration of the 1L treatment. *ESR1* alterations were systematically more prevalent in the AI+CDK4/6i cohort when splitting the post-group by duration of 1L treatment before sample collection (Fig. 2d). Second, we found that *ESR1* alterations were present in samples from both de novo and recurrent patients after 1L treatment (Supplementary Fig. 2b). It should be noted that *ESR1* alterations were also found in a few tumors sampled from patients with de novo metastatic disease prior to 1L. Because those patients may have been misclassified as de novo due to a gap in their clinical history, we cannot conclude that *ESR1* alterations may be present prior to treatment. Third, there were no substantial differences between patients with visceral disease vs. those without (*p* > 0.21). Last, we found that the prevalence of *ESR1* alterations in samples from the primary location was similar to the prevalence in metastatic samples (*p* > 0.26; Supplementary Fig. 2c). In addition, the Y537 and D538 activating *ESR1* mutations occurred in samples from the primary location as often as in metastatic samples following AI+CDK4/6i (Supplementary Fig. 2d). Those results suggest that lesions in the primary site can harbor *ESR1* oncogenic mutations after 1L treatment, addressing an ongoing discussion about the site of *ESR1* mutations^8,15,16,18,19,44,45^. To consolidate the stratified analysis amongst post-treatment groups, we performed a multivariate logistic regression model of *ESR1* occurrence in all treatment cohorts based on clinical variables. We identified “sample collection after exposure to AI and AI+CDK4/6i” as the only significant variable. When we performed a similar analysis directly comparing the post-AI vs. post-AI+CDK4/6i cohorts, we identified “1L duration over 1 year” (*p* < 1.6 × 10^−5^) and “CDK4/6i exposure” (contrast against “AI exposure only”, *p* = 5.4 × 10^−4^) as the two significant clinical variables (positive coefficients), whereas the interaction effect of “1L duration and CDK4/6i exposure” was not significant (see Methods). Those results confirmed that both “1L duration over 1 year” and “CDK4/6i exposure” contribute independently to a higher prevalence of *ESR1* alterations after 1L treatment.

### TP53 alteration is associated with shorter 1L duration

Beyond *ESR1*, we found that *TP53* alteration prevalence was also significantly higher after 1L (Fig. 2e): from 23.4% (CI: 18.3–28.9) pre-treatment to 36.2% (CI: 27.6-45.7) post-treatment in the AI cohort (*p* = 0.086); from 25.2% (CI: 21.5–29.1) to 39.4% (CI: 30.3–48.6) in the AI+CDK4/6i cohort (*p* = 0.002); and from 27.1% (CI: 22.0–31.2) to 39.6% (CI: 26.4–52.8) in the Fulv+CDK4/6i cohort (*p* = 0.040). It remained unchanged in the chemotherapy cohort. Prevalence of *TP53* alterations was associated with duration of the 1L treatment in both AI-based cohorts, similarly to the results in the adjuvant setting (Fig. 2f). It was highest in samples taken within the first year of 1L ( > 50%) and dropped to ~30% for samples taken later than 1 year after the start of 1L, which was close to pre-1L prevalence. We then performed a similar multivariate logistic regression model as explained above to understand clinical factors that drove *TP53* alteration occurrence in the AI-based cohorts. By comparing the post AI vs. AI+CDK4/6i cohorts, we identified “1L duration over 1 year” (*p* = 0.003) as the only significant clinical variable (negative coefficient), whereas “CDK4/6i exposure” as well as the interaction effect of “1L duration and CDK4/6i exposure” were not significant (see Methods). These results were consistent with an enrichment of *TP53* alterations in fast progressing tumors^40^ due to either intrinsic or rapidly acquired resistance, in contrast to *ESR1* alterations that are more likely to be acquired under treatment at a later time point. This observation post-1L was consistent with the result in the adjuvant setting (Fig. 2d, f).

### Multiple genes have significantly higher alteration prevalence following AI+CDK4/6i

**Fig. 3:**
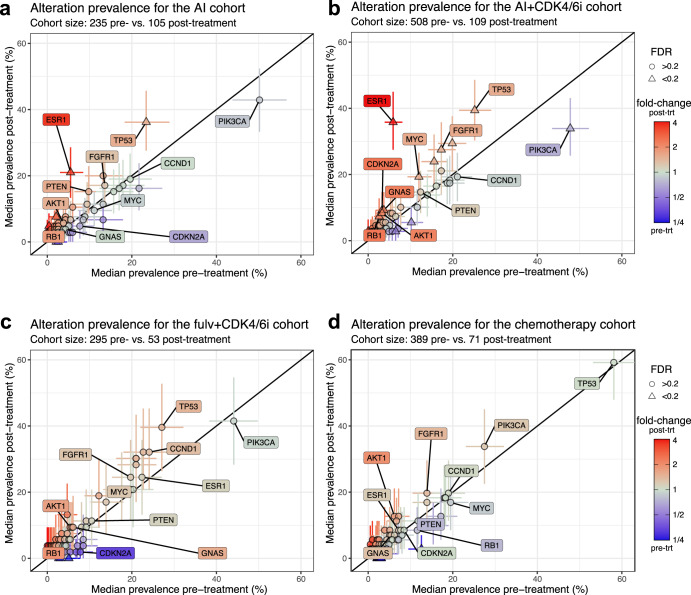
Prevalence of genomic alterations in tumors prior and after 1L treatment in the advanced setting. **a**–**d** Median prevalence of alterations prior to (x-axis), or after (y-axis) treatment for **a** AI therapies (**b**) AI+CDK4/6i therapies (**c**) fulvestrant + CDK4/6i therapies, or **d** chemotherapies. Each point is an individual gene; genes of interest are labeled. Error bars represent the 95% confidence interval; Color reflects fold-change; Shape significance with an FDR cutoff of 0.2 based on bootstrapping and Benjamini-Hochberg procedure.

We then systematically assessed changes in the tumor genomic landscape based on genes of the CGP panel (Fig. 3a–d). In the AI+CDK4/6i cohort specifically, we identified additional genes with significantly higher alteration prevalence post-treatment (Fig. 3b). *FGFR1* activating alterations were found in 27.5% (CI: 19.3-35.8) of samples post-treatment compared to 17.3% (CI: 14.2–20.7) pre-treatment (FDR = 0.17), reflecting a potential role in resistance to CDK4/6i^25,46–48^. *RB1* showed a 2.31-fold (CI: 1.0-4.61, FDR = 0.17) increase to 7.3% (CI: 2.8–12.8) post-treatment, consistent with the known impact of Rb-loss on CDK4/6i sensitivity^9,24,26,49^. A similar trend was present in the Fulv+CDK4/6i cohort (1.78-fold [CI: 0.29–4.56], FDR = 0.35), but at lower prevalence post-treatment (5.7%, [CI: 0.0–13.2]). Prevalence of *AKT1* alterations was increased 2.09-fold (CI: 0.98–3.92, FDR = 0.17) in the AI+CDK4/6i cohort as observed previously^4^. The same trend was observed in the other cohorts but was not significant, potentially due to low number of patients with *AKT1* alterations. Other genes with significant difference in the AI+CDK4/6i cohort comprised *MYC* (from 12.0% [CI: 9.3–15.0] to 19.3% [CI: 11.9–26.6], FDR = 0.17), *GNAS* (from 3.7% [CI: 2.2–5.5] to 9.2% [CI: 4.6-14.7], FDR = 0.17), and *CDKN2A* (from 3.3% [CI: 1.8–4.9] to 9.2% [CI: 4.6–14.7], FDR = 0.17). None of those genes had significantly higher alteration prevalence post-AI or post-chemotherapy, and the post-treatment prevalence was highest in the AI+CDK4/6i cohort, reinforcing the specific and profound impact of CDK4/6i on the tumor genomic landscape.

### Exclusivity of alterations after 1L therapies highlights multiple oncogenic pathways

Using DISCOVER^50^, we evaluated the mutual exclusivity of commonly altered genes and found that *ESR1* and *TP53* alterations are strongly exclusive in the post-AI group (*p* = 0.0086) as previously reported^8,16,43,51^, but less so after AI+CDK4/6i treatment (*p* = 0.044; Supplementary Fig. 3a). Additionally, we observed that *FGFR1* alterations were significantly exclusive of *PIK3CA* alterations (*p* = 0.0046 for post−AI+CDK4/6i) and more generally of other genes of the PI3K/AKT pathway (*PTEN* and *AKT1*)^3,16,19^ as well as *GNAS* (Supplementary Fig. 3b, *p* = 0.037 for the gene set post−AI+CDK4/6i). Our data identified two additional groups of mostly exclusive genes: *CDKN2A*/*RB1*/*CCND1* (Supplementary Fig. 3c) and *NF1*/*MAP3K1*/*MAP2K4* (Supplementary Fig. 3d). Based on these results, we defined five gene sets to perform pathway-level analyses: FGFR1/PI3K pathway, cell cycle, and MAPK pathway, as well as *ESR1* and *TP53* as individual genes. In the post-AI group, *ESR1* alterations were exclusive from MAPK pathway alterations as previously described (*p* = 0.003, Fisher’s exact test)^19,52^, but not in the post-AI+CDK4/6i cohort (*p* = 0.62, Supplementary Fig. 4a, b). Moreover, *ESR1* alterations in post-AI+CDK4/6i samples tended to be co-occurring with alterations in at least one other gene set (*p* = 0.092, Supplementary Fig. 4b) in contrast to the post-AI samples in which *ESR1* alterations are exclusive from alterations in other pathways (*p* = 0.032).

**Fig. 4:**
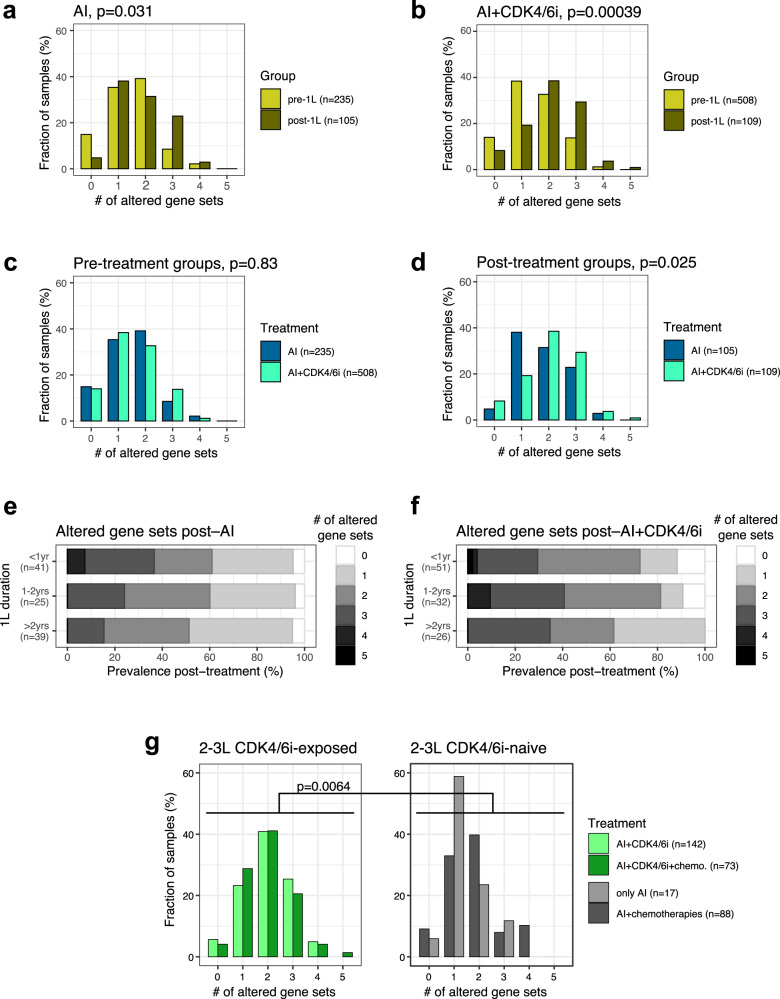
Prevalence of pathway-level alterations is increased in tumors exposed to CDK4/6i. **a**–**d** Distribution of the number of altered gene sets for pre- and post-treatment groups of the (**a**) AI, or **b** AI+CDK4/6i cohorts, as well as for the (**c**) pre-treatment and **d** post-treatment groups of AI-based cohorts. *P*-values based on a Kolmogorff-Smirnov test. **e**, **f** Distribution of the number of altered gene sets for the post-treatment samples of the (**e**) AI and **f** AI+CDK4/6i cohorts based on the 1L treatment duration prior to sample collection. **g** Distribution of the number of altered gene sets of samples collected after 2L or 3L based on treatment cohorts. *P*-value between samples exposed to CDK4/6i (left) or not (right) based on a Kolmogorff-Smirnov test.

Based on alterations in genes included in the five gene sets defined above, we assessed the number of sets with altered genes in individual samples. We found a significant increase in the number of concomitant altered gene sets following AI and AI+CDK4/6i treatments (*p* = 0.031 and, respectively, *p* = 3.9 × 10^−4^, one-sided Kolmogoroff-Smirnoff test, Fig. 4a, b). This increase was less pronounced in the Fulv+CDK4/6i (*p* = 0.13) or chemotherapy (*p* = 0.097) cohorts (Supplementary Fig. 4c, d). The number of concomitant altered gene sets for the AI-based cohorts was similarly distributed pre-1L (*p* = 0.83, Fig. 4c), but increased to significantly higher values in the post-AI+CDK4/6i samples compared to post-AI ones (*p* = 0.025, Fig. 4d). Indeed, 72.5% of CDK4/6i-treated samples had two or more altered gene sets versus only 57.1% of the AI-treated samples. That difference was systematic across different 1L durations with a peak of 33.9% of samples taken between 1 and 2 years of AI+CDK4/6i treatment having alterations in at least 3 gene sets (Fig. 4e, f). Therefore, concomitant alterations in multiple oncogenic pathways were most common in tumor samples after CDK4/6i exposure compared with samples without CDK4/6i exposure.

### Genomic landscape of tumors after 2L treatment confirms 1L results

Next, we focused on tumor samples from patients exposed to multiple lines of therapy in the advanced setting. Prevalence of *ESR1* alterations in samples collected after 2L confirmed the association with CDK4/6i exposure that we observed after 1L: for patients treated with AI or AI+chemotherapies, *ESR1* alteration prevalence was around 15-30%, whereas cohorts of patients exposed to AI+CDK4/6i had a prevalence around 30-40% (Supplementary Fig. 5a). It should be noted that Tumor mutational burden (TMB) was low and similar across samples from all cohorts (Supplementary Fig. 5b). Further, in these cohorts, the prevalence of *ESR1* mutations in samples from the primary location remained comparable to the one from metastatic site samples (Supplementary Fig. 5c). In addition, the number of concomitant altered gene sets remained higher in samples exposed to AI+CDK4/6i compared to those only exposed to AI or AI+chemotherapies after the 2–3L treatment (*p* = 0.0064, Fig. 4g).

### Clinical trial data confirm the impact of CDK4/6i on tumor genomic landscape

**Fig. 5:**
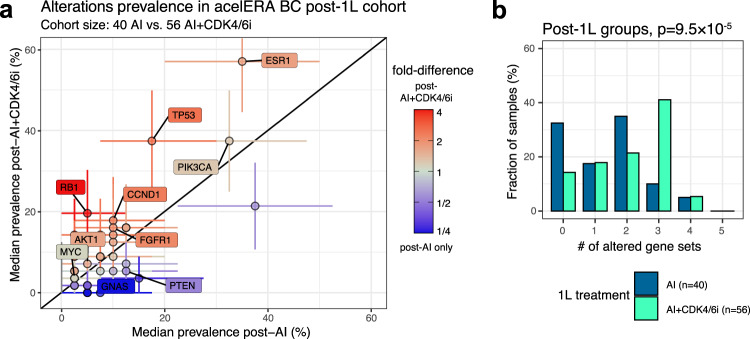
Prevalence of genomic alterations is higher for tumors from post-1L patients exposed to CDK4/6i in the acelERA trial. **a** Median prevalence of alterations in samples from post-1L patients who received AI (x-axis) or AI+CDK4/6i (y-axis) as 1L treatment. Each point is an individual gene; genes of interest are labeled. Error bars represent the 95% confidence interval; Color reflects fold-change; Shape significance with an FDR cutoff of 0.2 based on bootstrapping and Benjamini-Hochberg procedure. **b** Distribution of the number of altered gene sets for the samples from post-1L patients who received AI or AI+CDK4/6i as 1L treatment. *P*-values based on a Kolmogorff-Smirnov test.

To validate the results obtained from RWD, we leveraged the liquid biopsy (LB)-based CGP data collected at baseline for a recent clinical trial, acelERA BC (NCT04576455) that enrolled 2L/3L patients with ER+/HER2- locally advanced BC or mBC^41^. It should be noted that the LB-based CGP assay has a different sensitivity than the CGP assay for solid tumors and may potentially identify alterations from multiple lesions. Because of this difference we limited the analysis to comparisons within the acelERA BC trial data. Among the 2L patients, 40 had an AI therapy as 1L, whereas 56 had AI+CDK4/6i as 1L. Samples from patients of the AI+CDK4/6i cohort had significantly higher *ESR1* (fold-difference of 1.64 [CI: 1.05-2.85], *p* = 0.016) and *TP53* alteration prevalence (fold-difference of 2.14 [CI: 1.09-5.95], *p* = 0.013) compared to samples from the AI-only cohort (Fig. 5a). Alterations of the cell cycle (*RB1* mostly) and MAPK gene sets were also more prevalent in samples of the AI+CDK4/6i cohort. The overall number of altered gene sets as defined above was also significantly higher (*p* = 9.5 × 10^−5^, Fig. 5b). In particular 67% of samples post-AI+CDK4/6i had at least two altered gene sets in comparison to 50% for the post-AI cohort. When we expanded our cohorts to comprise post-2L patients and those exposed to chemotherapies (*N* = 80 patients treated with an AI+CDK4/6i regimen in 1L or 2L; *N* = 75 patients not exposed to CDK4/6i), the results were qualitatively similar with *ESR1* and *RB1* being significantly higher post-CDK4/6i (Supplementary Fig. 6). Thus, clinical trial data confirmed the impact of CDK4/6i on the genomic profile of HR+/HER2- tumors we observed in RWD.

## Discussion

Approval of CDK4/6i has dramatically changed the therapeutic landscape of HR+/HER2- mBC, and recent clinical trials in the adjuvant setting are further shaping patient care^53^. The next class of drugs that may be practice-changing is oral SERDs, whose therapeutic benefits over AI and fulvestrant may be stronger for advanced patients with *ESR1*-mutated tumors^33,34,41,54^. In order to optimally develop and use those therapies, it has become critical to understand how exposure to CDK4/6i changes the genomic landscape of metastatic tumors, in particular regarding mutations associated with treatment outcomes such as *TP53* and *ESR1*. Our results based on RWD, and validated using clinical trial data, offer five conclusions to that question. First, the exclusivity between *TP53* and *ESR1* mutations is reflected in the duration of the adjuvant and 1L treatments. In both settings, patients whose tumor progressed within 1 year on ET are more likely to harbor *TP53* mutations whereas resistant tumors treated with >1 year on ET are enriched in *ESR1* alterations. The differences between *TP53* and *ESR1* alteration prevalence are likely due to an earlier progression of tumors with prior *TP53* alterations, whereas *ESR1* mutations are more likely to be acquired in tumors initially responding to treatment. Second, prevalence of *ESR1* alterations is significantly higher following AI+CDK4/6i exposure compared to AI alone. This result was consistent across stratified analyses, including duration of 1L treatment, and validated in clinical trial data. Third, beyond *ESR1*, other genes are more often altered after AI+CDK4/6i exposure compared to AI alone. *FGFR1* was of interest due to previous reports on its role in CDK4/6i resistance^25,46–48^. Its exclusivity with alterations of the PI3K/AKT pathway suggested functional redundancy in overcoming AI+CDK4/6i^27,29,50,55^. *GNAS* is less known but may be meaningful given its association with poor prognosis^37^ and exclusivity with other genes in the PI3K/AKT pathway. The increase in *RB1* alterations further support the role of *RB1* loss of function in progression on CDK4/6i^9,26,56^. It should be noted that the CGP assay leveraged here captures only genomic alterations and not changes in gene expression. This may be relevant for *CCNE1* and *CDK6* whose up-regulation has been associated with progression on CDK4/6i in BC^28,56^. Fourth, tumors post-AI+CDK4/6i showed concomitant occurrence of alterations in multiple oncogenic pathways. In particular, *ESR1* and *TP53* alterations are more likely to occur with alterations in genes of other pathways (cell cycle, PI3K/AKT, or MAPK) in tumors exposed to CDK4/6i compared to those exposed only to AI. Finally, the effects of CDK4/6i exposure are still observed in later line samples, suggesting that CDK4/6i treatment may have a lasting effect on the HR+/HER2- tumor genomic profile.

As our work relied on retrospectively collected RWD, we identified and addressed several caveats due to the nature of the data and the questions we asked in this work. First, our study is not based on paired samples, which is the gold standard to study resistance mechanisms. Our method, relying on comparing cohorts defined by the date of sample collection relative to treatment, is aiming at addressing this challenge intrinsic to secondary usage of data (in this case RWD) not purposely collected to study resistance mechanisms. While not explicitly identifying resistance mechanisms, our results allowed us to describe the evolving genomic landscape of patients’s tumors across lines of therapies. Second, completeness of the data from routine clinical practice may vary, which can weaken the signal for comparisons based on patient clinical history. For example, prior adjuvant treatments for some metastatic patients may not be reported in the database and those patients may have thus been misclassified as de novo. This caveat is outweighed by the larger number of patients in our dataset which allowed us to identify significant signals in RWD. Last, comparison groups can be biased, which can lead to false positive results. For example, samples used for CGP were more likely coming from the primary location in the pre-treatment group whereas the post-treatment group contained more samples from metastatic sites. Our approach to address this issue was to perform stratified analysis based on potential confounders, build multivariate models to identify significant contributors to the observed signal, and bootstrap our results and correct for multiple testing to assess significance. Our results showed no qualitative differences based on clinical variables and were robust through multiple updates of the data cut. Our stratified analyses and conservative interpretation of the results were designed to exclude potential confounders of the signal observed in the overall cohort and thus avoid false positive results. We therefore found that the challenges in studying resistance mechanisms from retrospectively collected RWD can be addressed by the rigorous approach we proposed and thus allowed to leverage a broader and larger patient population than the one traditionally found in clinical trial data to identify significant differences in tumor genomic profiles.

We anticipate multiple translational outcomes of our findings. First, our results identified that the duration of AI treatment is associated with a different genomic profile: tumors from patients relapsing between 1 and 2 years of adjuvant ET treatment (currently classified as primary ETR) have a genomic profile more similar to the profile of tumors from patients with secondary ETR than those relapsing within 1 year. In the advanced setting, tumor profiles also differ based on a 1-year cutoff for 1L duration. Thus, stratification of patients by duration of adjuvant and 1L therapies may allow for selection of tumors with different biologies. Second, the increase in *ESR1* mutation prevalence following CDK4/6i exposure will make the role of oral SERDs important in inhibiting mutant *ESR1* activity given CDK4/6i-based regimens have become the standard of care in the metastatic setting. Third, upon progression on CDK4/6i, multiple oncogenic pathways are likely to be mutated in the same tumor, stressing the need for novel therapies that can combine with oral SERDs and cell cycle inhibitors with tolerable toxicity. In parallel to PI3K/AKT pathway inhibitors and other therapies targeting mutations in the MAPK pathway or *FGFR1* amplification, the development of antibody-drug conjugate therapies may address this need^57^. Finally, the development of new therapies for patients with advanced HR+/HER2- BC will have to account for a genomic landscape that may further evolve with the approval of CDK4/6i in the adjuvant setting^53,58^.

In conclusion, analysis of RWD linked to comprehensive genomic profiling can uncover differences in the tumor genomic landscape associated with treatment regimens. Bootstrapping, stratified analysis, and comparison with clinical trial data reinforced our confidence in those results and thus allowed us to identify that CDK4/6i exposure led to a different—more altered—genomic landscape of HR+/HER2- BC tumors. This result and the association of *ESR1* alteration prevalence with the time under treatment can inform design of clinical trials in the metastatic setting and may help guide treatment strategy for advanced patients. Beyond the implications for patient care and drug development, our work demonstrates the feasibility of leveraging real-world clinico-genomic data for translational research in oncology and the identification of more personalized treatment strategies.

## Methods

### Cohort inclusion criteria

The FH database is a nationwide, USA-based, retrospective longitudinal database, comprising de-identified patient-level structured and unstructured data, curated via technology-enabled abstraction^36,42^. The FH-FMI CGDB is a de-identified database linking the FH population de-identified EHR-derived data to genomic data derived from FMI CGP tests by de-identified, deterministic matching^36^. Patients were included in the breast cancer (BC) cohort of FH-FMI CGDB if: (1) they had at least two documented clinical visits in the FH Network, on different days, occurring on or after January 1, 2011; (2) they had been diagnosed with BC (based on the International Classification of Diseases Ninth Revision—Clinical Modification [ICD-9—CM] codes: ICD-9 174.x [malignant neoplasm of the breast] or 175.x [malignant neoplasm of male breast], or the International Classification of Diseases Tenth Revision—Clinical Modification [ICD-10—CM] code: ICD-10 C50x [malignant neoplasm of breast]); (3) they had undergone CGP testing by an FMI test on a sample with a pathologist-confirmed histology that was consistent with BC; (4) they had undergone CGP testing with report date and specimen collection date no earlier than 30 days before, on, or at any time after the FH chart-confirmed date of initial diagnosis of BC (if the specimen collection date was not available, only the FMI report date had to meet this criteria; if the initial diagnosis date was not available, the earlier of the patient’s locoregional recurrence or metastatic BC diagnosis date was used); (5) their demographic information was available at FH and their FMI testing report was uniquely and deterministically matched by a third-party linking vendor; and (6) FH chart-confirmed diagnosis of BC was made on or after January 1, 2011. Patient clinical data and genomic data from biopsy samples from these patients were collected as described before^36,42,59^.

BC subtype group was determined based on immunohistochemistry and fluorescent in situ hybridization test results of estrogen receptor-, progesterone receptor-, and HER2-status as documented within the EHR. Patients were considered to have HR-positive disease if their tumors were estrogen receptor- and/or progesterone receptor-positive. Patients were considered to have HER2-positive disease if their tumors were recorded as fluorescent in situ hybridization-positive/amplified, immunohistochemistry-positive (3+), or positive NOS. For HR/HER2 status, a patient was considered positive for a biomarker if the status of a given biomarker within the 90-day window before or after mBC diagnosis was positive. If unavailable within a 90-day window, eBC testing data was used in replacement of mBC testing data to determine biomarker status We applied the following hierarchy to the BC subtype biomarker assessment: positive > negative > equivocal > unknown. HR-positive, HER2-negative, subtype was defined as patients with HR status as positive and HER2 status as negative or equivocal.

### Tumor genotyping

Genomic alterations were identified via CGP of >300 cancer-related genes on FMI’s next-generation sequencing (NGS) test (FoundationOne®CDx, FoundationOne®)^36,42,59^ which are both based on solid tumor biopsy. The classification into samples from the primary versus metastatic locations is based on the site in the body from which the assayed tumor material was extracted. For most solid tissue specimens, the free-text information provided in the Specimen Site field on the test requisition form can be mapped to a controlled vocabulary. Data from liquid CGP testing were insufficient in patient number to present meaningful results from the CGDB. For the acelERA BC trial, 229 patients (out of 303 enrolled) had data available from CGP performed at baseline using FMI’s ctDNA test (FoundationOne® Liquid CDx) which is based on liquid biopsy.

Alterations (copy number variation, point mutations, and rearrangements) were categorized as either known pathogenic, likely pathogenic, or variants of unknown significance. For this work, we considered as ‘altered’ only the known and likely pathogenic alterations independently of their nature. Germline single-nucleotide polymorphisms were ignored in our analysis. Tumor mutational burden (TMB), a measure of the number of somatic mutations identified per megabase of DNA sequenced, was calculated for most samples^60^.

### Statistical analysis of differences in alteration prevalence

After defining cohorts based on treatment, we estimated for each gene the changes in prevalence of alterations between pre-treatment and post-treatment groups or between cohorts. This estimator is akin to the risk ratio of proportions, which we referred to as *fold-change ratio* to reflect the design where the calculation is between pre/post-groups of the same treatment cohort and interpreted as *change* from pre- to post-treatment potentially due to treatment interaction, or as *fold-difference ratio* to reflect the design when calculation is over groups from different treatment cohorts. To estimate this ratio, as well as the *prevalence* of alterations in the groups (proportions), we used non-parametric bootstrap methods. We also calculated the percentile confidence intervals with an *alpha* = *0.05* as well as an empirical *p*-value for the null hypothesis that *fold-change ratio* = *1*. Statistical tests were 2-sided. When performing a systematic analysis of all genes in the CGP, *p*-values thus calculated is then corrected for multiple testing using the Benjamini-Hochberg procedure for all the genes tested in the panel with an alteration prevalence above 2% (either pre- or post-group) for a given comparison (see supplemental data files). We considered *p*-values of less than 0.05 as statistically significant and an FDR of 0.2 as an acceptable cutoff given the exploratory nature of these experiments as well as downstream use in decision-making. Whereas strong and significant increases in prevalence are most likely to be related to treatment, it is worth noting that the interpretation of marginal increase or decrease in prevalence post-treatment can be ambiguous, even if significant, due to patient selection bias.

### Stratified analysis and logistic regression to assess potential confounders

As explained above, our primary metric of association of genomic changes with treatment is the crude fold-change or fold-difference ratio calculated over a complete cohort. To assess effects of potential confounders of this association, we performed stratified analysis by defining strata based on the patient characteristics and sample properties (Fig. 1d). We considered the following potential confounders of alteration prevalence: duration of 1L prior to post-treatment sample collection, de novo vs. recurrent disease, sample location (primary vs. metastatic), presence or absence of visceral metastases, and patient race. Our approach was to qualitatively compare the metric by stratification into sub-cohorts, controlling for each potential confounder of alteration prevalence one at a time. If the crude fold-change metric is similar to stratum-specific metrics, the association of treatment with genomic changes is robust to patient selection and confounding factors.

To confirm our findings, we performed logistic regression to identify clinical factors driving alteration prevalence. For the pre-post group comparisons, we regressed on the potential confounders listed above and an additional binary variable for the sample group (pre- or post-treatment). If the sample timing variable is the only significant variable, it confirms that none of the confounders between pre-post groups explain the difference in alteration prevalence. For other cohort comparisons, we regressed on potential confounders and an additional variable that captured the difference in cohorts (e.g. “treatment for AI” vs. “AI+CDK4/6i comparison”) and interaction terms where it was relevant (e.g. “treatment regimen and duration of 1L treatment”).

### Exclusivity analysis and its significance

We used the method DISCOVER^50^ to identify groups of mutually exclusive genes. Group-wise or pair-wise tests were performed on the set of genes of interest. For the pathway-level analysis, we used the Fisher’s exact test to identify mutually exclusive pairs of gene sets.

### Reporting summary

Further information on research design is available in the Nature Research Reporting Summary linked to this article.

### Supplementary information

Supplemental figures 1–5 + table

Reporting summary

## Data Availability

Alteration prevalence in the pre-1L and post-1L groups for genes with an alteration prevalence above 2% and related statistics for each cohort are available in the supplementary data files. The raw data that support the findings of this study have been originated by Flatiron Health, Inc. and Foundation Medicine, Inc. These de-identified data may be made available upon request, and are subject to a license agreement with Flatiron Health and Foundation Medicine; interested researchers should contact <cgdb-fmi@flatiron.com> and <dataaccess@flatiron.com> to determine licensing terms.

## Code availability

Analyses were performed using the R software version 4.2.0 (R Foundation for Statistical Computing) and customized scripts. Code for data analysis can be requested from the corresponding author.
