# Supplementary material for: CDK4/6i-treated HR+/HER2- breast cancer tumors show higher ESR1 mutation prevalence and more altered genomic landscape
Source: NPJ Breast Cancer. 2024 Feb 22;10:15. doi: 10.1038/s41523-024-00617-7 (PMC10883990; doi:10.1038/s41523-024-00617-7)
Supplement: Supplementary file 1 — Supplemental figures 1–5 + table [file 41523_2024_617_MOESM1_ESM.pdf]

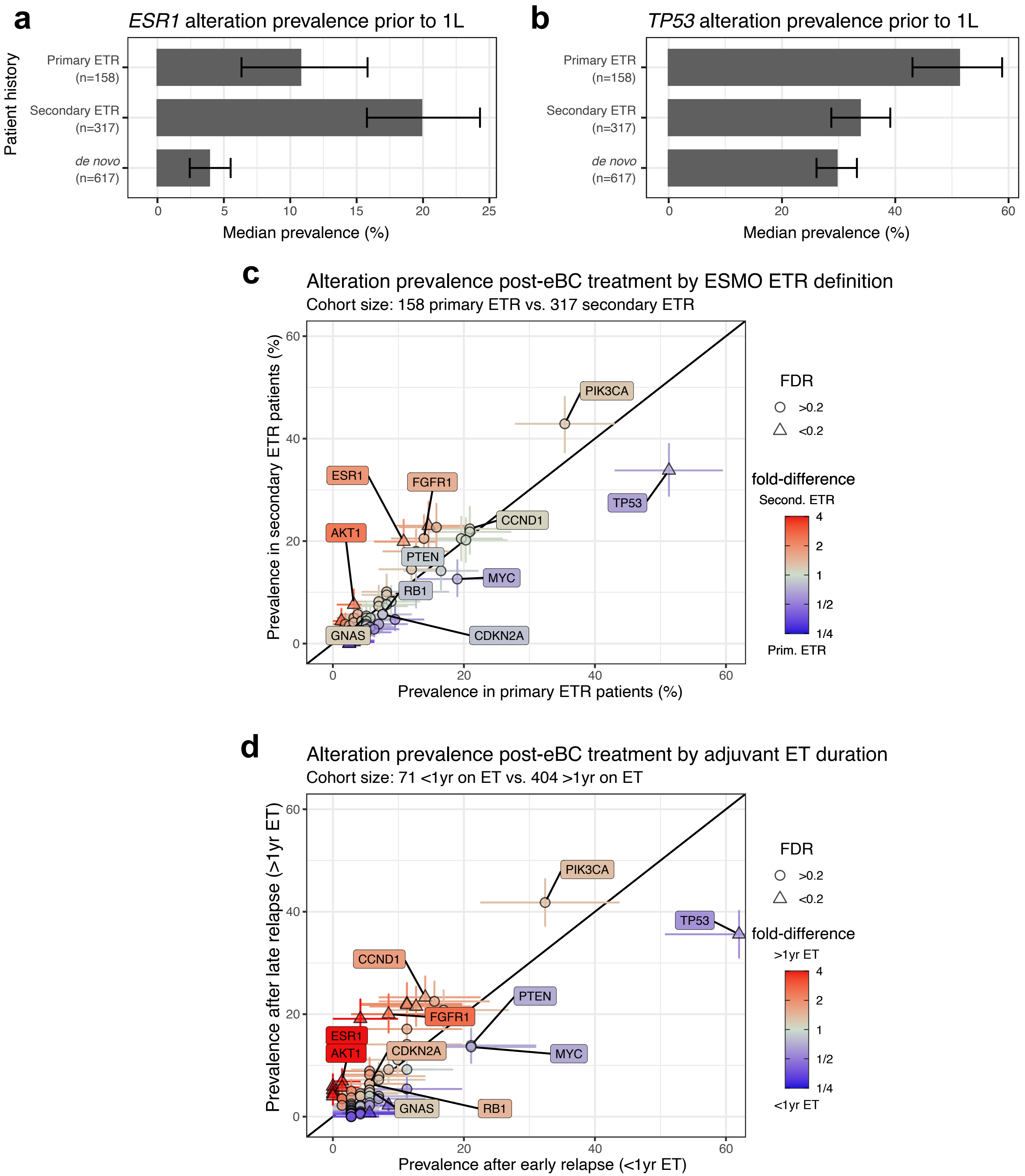

Supplementary Figure 1: Prevalence of genomic alterations in tumors from recurrent patients. **(a-b)** Prevalence of *ESR1* (a) and *TP53* (b) alterations in samples collected from patients based on adjuvant treatment. Error bars represent the 95% confidence interval based on bootstrapping. **(c)** Median prevalence of alterations in samples from patients with primary ETR (x-axis) or secondary ETR (y-axis). Each point is an individual gene; some genes of interest are labeled. Error bars represent the 95% confidence interval; Color reflects fold-change; Shape significance with an FDR cutoff of 0.2 based on bootstrapping and Benjamini-Hochberg procedure. **(d)** Same as (c) with x-axis based on tumor samples from patients who replased within one year of adjuvant ET and y-axis based on tumor samples from patients who replased after more than one year of adjuvant ET.

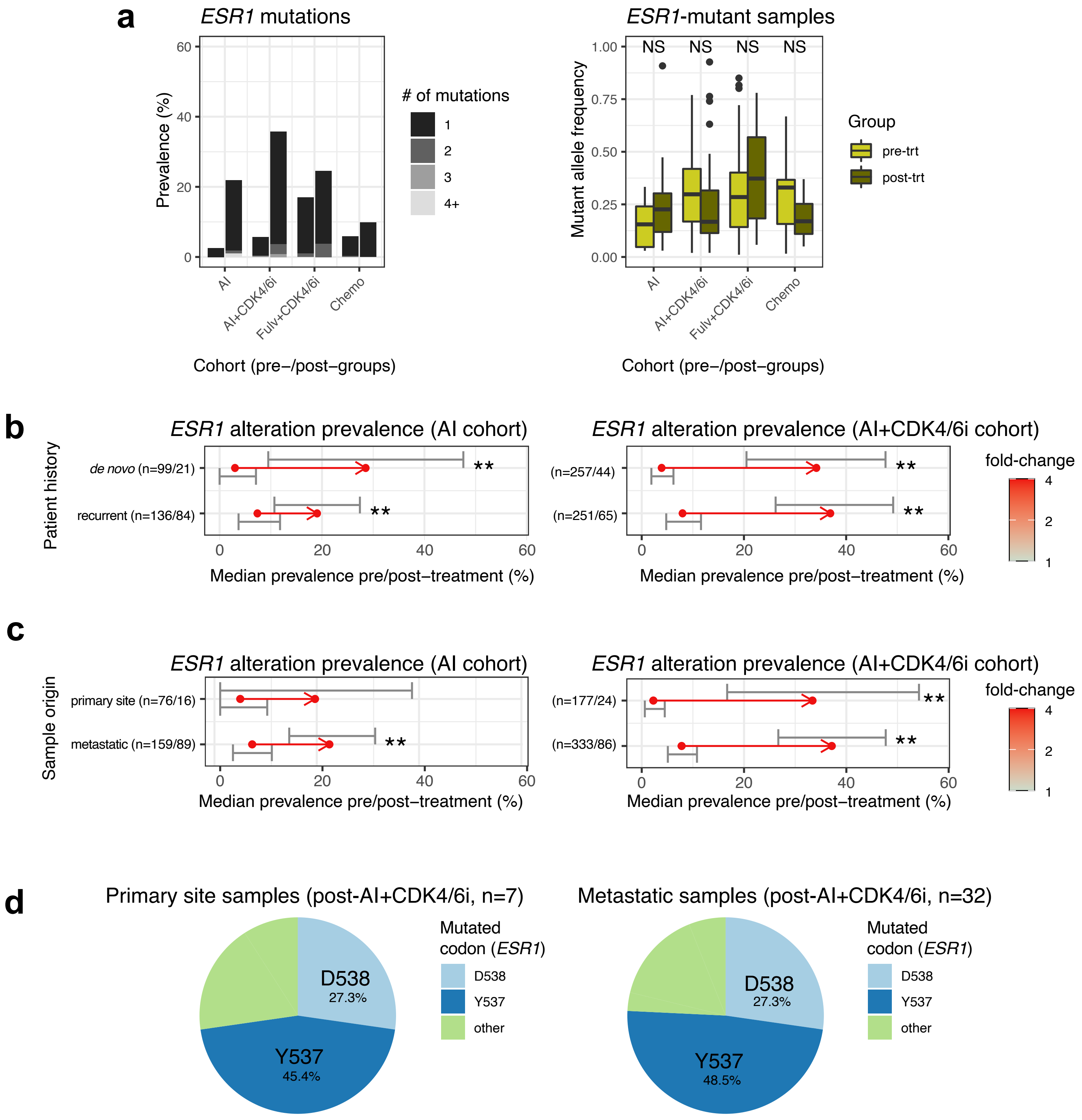

Supplementary Figure 2: Allele frequency and stratified analysis for *ESR1* alterations in 1L cohorts. **(a)** Prevalence of samples with one or multiple *ESR1* mutations with frequency above 1% (left) and cumulative allele frequency of variants (right) for different cohorts and groups. NS stands for P≥0.05, based on Wilcoxon's rank sum test. Center line is the median; box is the first and third quartiles; whisker extends to the values no further than 1.5 * IQR; outliers are plotted individually. **(b-c)** Stratified analysis for *ESR1* alteration prevalence based on patient history (b) and sample origin (c) for the AI and AI+CDK4/6i cohorts. Arrows represent the difference in prevalence: origin is the median prevalence pre-treatment and end is the median prevalence post-treatment. Error bars represent the 95% confidence interval; Color represents fold-change magnitude; * stands for $p<0.05$, ** for $p<0.01$ based on bootstrapping. Strata are labeled on the y-axis. **(d)** Codon-level mutations in ESR1 with allele frequency above 1% for the samples for the post-AI+CDK4/6i group split by sample origin. Note that some samples have multiple mutations reported in the pie chart.

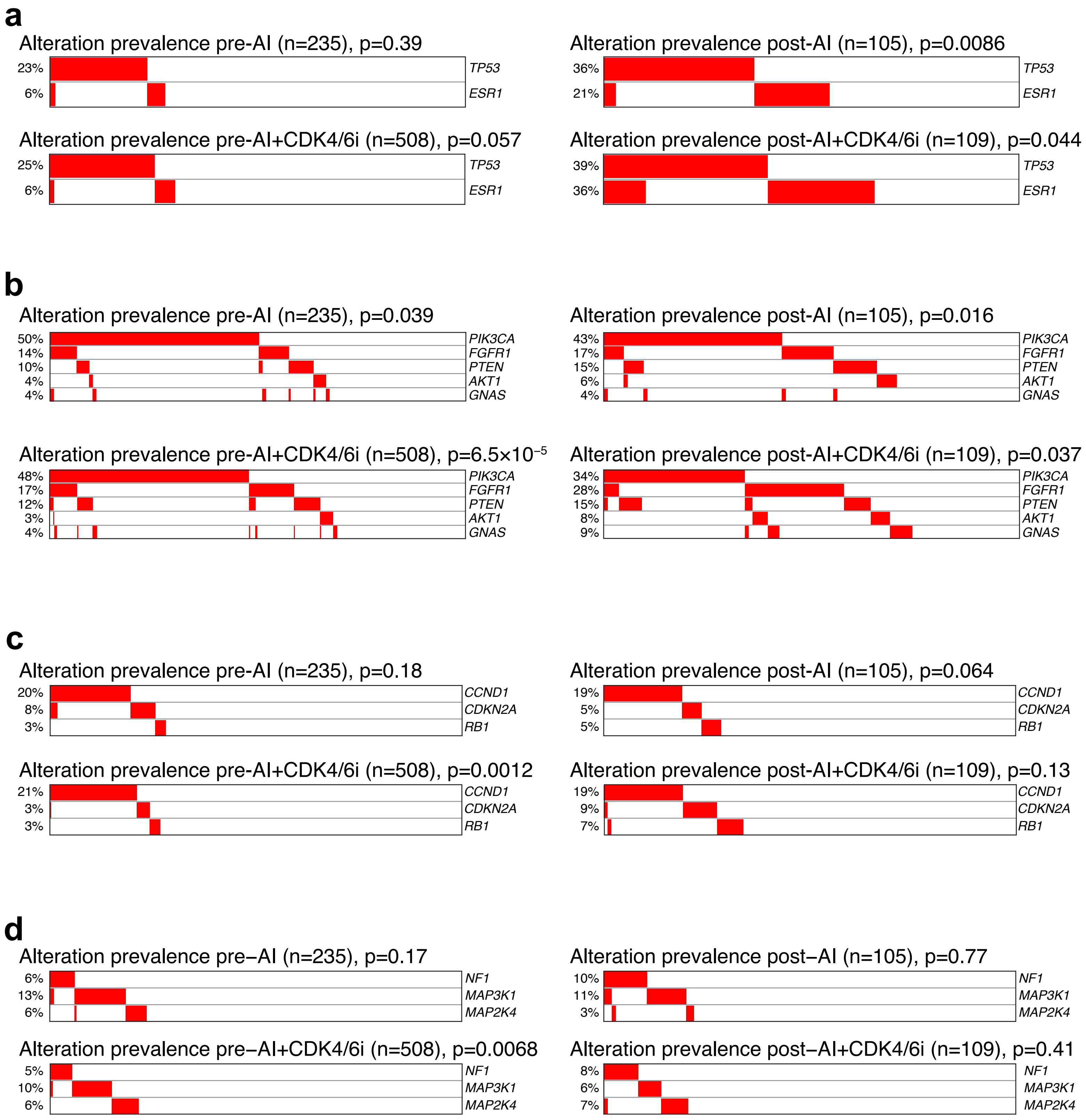

Supplementary Figure 3: Oncoprint plots for different sets of genes, treatment cohorts, and pre-/post-groups. **(a)** Prevalence of *TP53* and *ESR1* alterations in patients from the AI-based cohorts. **(b)** Prevalence of alterations in *FGFR1*, *GNAS*, and genes of the PI3K/AKT pathway in patients from the AI-based cohorts. **(c)** Prevalence of alterations in selected cell cycle genes in patients from the AI-based cohorts. **(d)** Prevalence of alterations in selected genes of the MAPK pathway from the AI-based cohorts. P-value calculated using DISCOVER for groupwise or pairwise exclusivity.

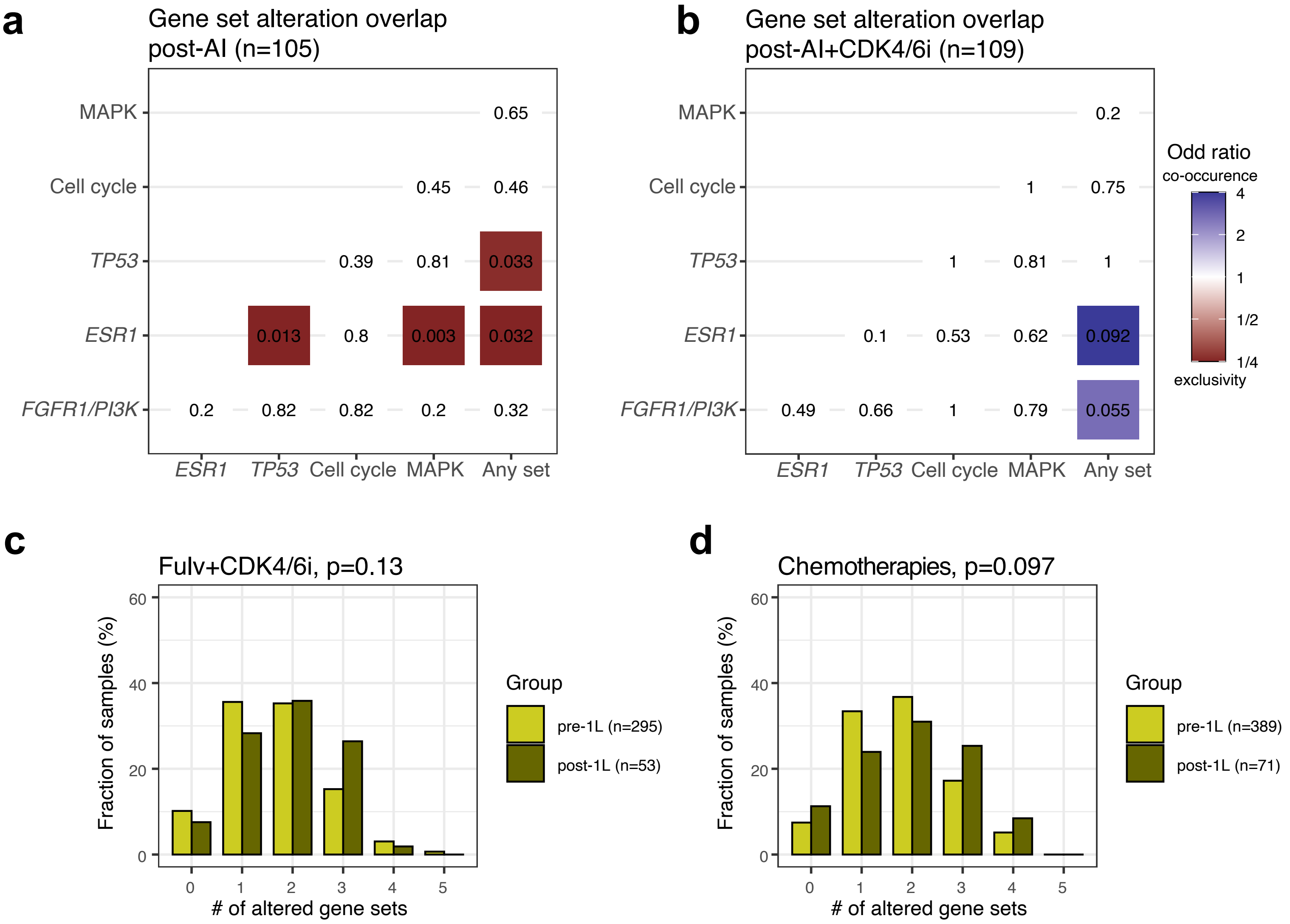

Supplementary Figure 4: Exclusivity of alterations in patient samples and prevalence of pathway-level alterations. **(a-b)** Exclusivity of pathway alterations post-treatment for the AI (a) and AI+CDK4/6i (b) cohorts. Numbers are p-values based on Fisher's exact test. Color represents the odd-ratio for co-occurence or exclusivity with $p<0.1$. **(c-d)** Distribution of the number of altered gene sets for pre- and post-treatment groups of the (c) fulvestrant+CDK4/6i and (d) chemotherapy cohorts. P-values based on a Kolmogorff-Smirnov test.

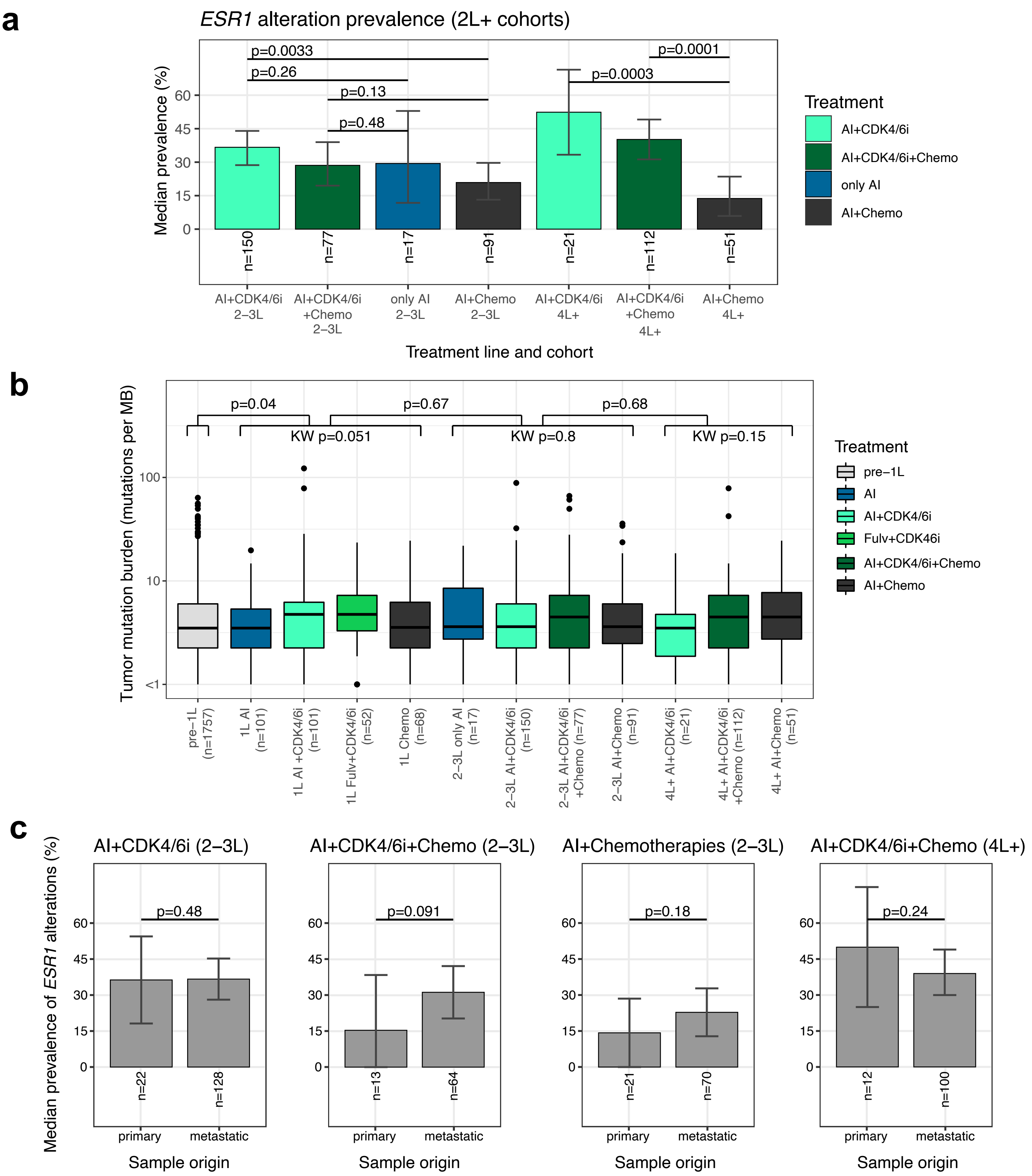

Supplementary Figure 5: Prevalence of genomic alterations in tumors after 2L treatment.
(a) Prevalence of ESR1 alteration in late-stage patients split by cohort and treatment line. P-values based on bootstrapping. **(b)** Tumor mutation burden (as mutations per MB) for the different cohorts and lines of treatments. P-values are based on a Wilcoxon's rank sum test for comparisons between lines of treatment or a Kruskal-Wallis (KW) test for comparisons between cohorts of the same line of treatment. Center line is the median; box is the first and third quartiles; whisker extends to the values no further than 1.5 * IQR; outliers are plotted individually. **(c)** Prevalence of *ESR1* alteration in samples from primary or metastatic locations in different cohorts. P-values based on bootstrapping. Error bars represent the 95% confidence interval for all plots.

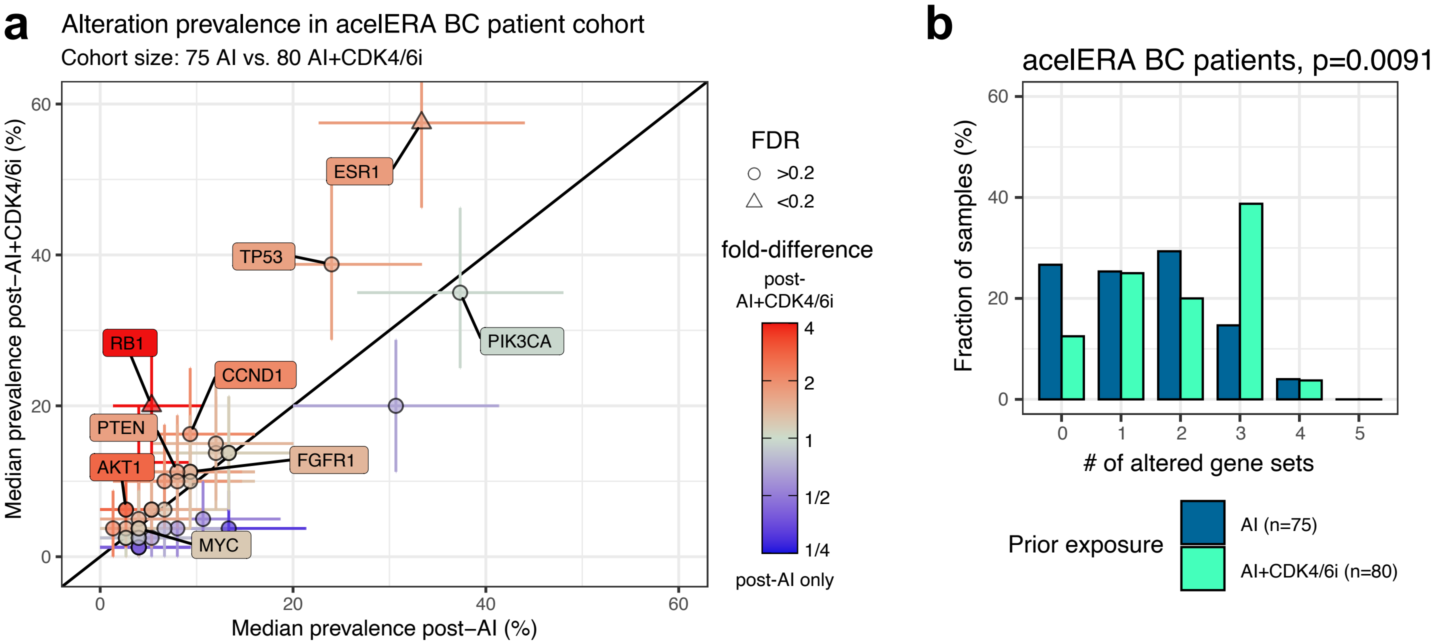

Supplementary Figure 6: Prevalence of genomic alterations in all acelERA trial patients.
(a) Median prevalence of alterations in samples from patients who received AI+CDK4/6i (y-axis) as 1L or 2L treatment vs. those who did not receive CDK4/6i (x-axis). Each point is an individual gene; genes of interest are labeled. Error bars represent the 95% confidence interval. Color reflects fold-change; Shape significance with an FDR cutoff of 0.2 based on bootstrapping and Benjamini-Hochberg procedure. **(b)** Distribution of the number of altered gene sets for the samples from patients who received AI+CDK4/6i as 1L or 2L treatment vs. those who did not receive CDK4/6i. P-values based on a Kolmogorff-Smirnov test.

	only AI (2-3L) (n=17)	AI+CDK4/6i (2-3L) (n=150)	AI+CDK4/6i +Chemo (2-3L) (n=77)	AI+Chemo (2-3L) (n=91)	AI+CDK4/6i (4L+) (n=21)	AI+CDK4/6i +Chemo (4L+) (n=112)	AI+Chemo (4L+) (n=51)
**Age at sample collection**							
Mean (SD)	64.5 (7.75)	62.1 (11.8)	59.1 (11.1)	59.8 (9.94)	60.5 (15.8)	59.0 (12.4)	58.3 (12.3)
Median [Min, Max]	63.0 [55.0, 82.0]	62.5 [33.0, 85.0]	59.0 [36.0, 82.0]	60.0 [32.0, 85.0]	64.0 [32.0, 81.0]	59.0 [32.0, 84.0]	58.0 [35.0, 85.0]
**Race**							
African American	0 (0%)	11 (7.3%)	8 (10.4%)	5 (5.5%)	1 (4.8%)	8 (7.1%)	2 (3.9%)
White	16 (94.1%)	116 (77.3%)	58 (75.3%)	68 (74.7%)	17 (81.0%)	77 (68.8%)	36 (70.6%)
Other*	1 (5.9%)	23 (15.3%)	11 (14.3%)	18 (19.8%)	3 (14.3%)	27 (24.1%)	13 (25.5%)
**Stage at met diagnosis**							
De-novo	2 (11.8%)	57 (38.0%)	28 (36.4%)	32 (35.2%)	9 (42.9%)	42 (37.5%)	13 (25.5%)
Recurrent	15 (88.2%)	93 (62.0%)	49 (63.6%)	59 (64.8%)	12 (57.1%)	70 (62.5%)	38 (74.5%)
**Sample collection site**							
Primary	1 (5.9%)	22 (14.7%)	13 (16.9%)	21 (23.1%)	2 (9.5%)	12 (10.7%)	4 (7.8%)
Metastatic	16 (94.1%)	128 (85.3%)	64 (83.1%)	70 (76.9%)	19 (90.5%)	100 (89.3%)	47 (92.2%)
**Tissue of origin**							
Breast	1 (5.9%)	22 (14.7%)	13 (16.9%)	21 (23.1%)	2 (9.5%)	12 (10.7%)	4 (7.8%)
Bone	4 (23.5%)	11 (7.3%)	5 (6.5%)	3 (3.3%)	2 (9.5%)	4 (3.6%)	6 (11.8%)
Liver	2 (11.8%)	50 (33.3%)	32 (41.6%)	27 (29.7%)	7 (33.3%)	56 (50.0%)	14 (27.5%)
Lung	1 (5.9%)	5 (3.3%)	1 (1.3%)	3 (3.3%)	0 (0%)	3 (2.7%)	2 (3.9%)
Lymph node	0 (0%)	17 (11.3%)	9 (11.7%)	11 (12.1%)	2 (9.5%)	9 (8.0%)	6 (11.8%)
Soft tissue	2 (11.8%)	9 (6.0%)	4 (5.2%)	9 (9.9%)	1 (4.8%)	2 (1.8%)	2 (3.9%)
Other	7 (41.2%)	36 (24.0%)	13 (16.9%)	17 (18.7%)	7 (33.3%)	26 (23.2%)	17 (33.3%)
**ECOG† performance status at sample collection**							
0	12 (70.6%)	58 (38.7%)	31 (40.3%)	39 (42.9%)	10 (47.6%)	36 (32.1%)	22 (43.1%)
1	2 (11.8%)	62 (41.3%)	26 (33.8%)	29 (31.9%)	8 (38.1%)	51 (45.5%)	18 (35.3%)
>=2	1 (5.9%)	10 (6.7%)	9 (11.7%)	8 (8.8%)	0 (0%)	11 (9.8%)	4 (7.8%)
Missing	2 (11.8%)	20 (13.3%)	11 (14.3%)	15 (16.5%)	3 (14.3%)	14 (12.5%)	7 (13.7%)
**Visceral disease at sample collection**							
Yes	5 (29.4%)	73 (48.7%)	53 (68.8%)	48 (52.7%)	8 (38.1%)	93 (83.0%)	27 (52.9%)
No	12 (70.6%)	77 (51.3%)	24 (31.2%)	43 (47.3%)	13 (61.9%)	19 (17.0%)	24 (47.1%)

* includes Unknown and missing values

† Eastern Cooperative Oncology Group

Supplementary Table 1: Sociodemographic and tumor characteristics for the second and later line patient cohorts
